# An age-period-cohort analysis of female breast cancer mortality from 1990–2009 in China

**DOI:** 10.1186/s12939-015-0211-x

**Published:** 2015-09-14

**Authors:** Chunhui Li, Chuanhua Yu, Peigang Wang

**Affiliations:** Department of Epidemiology and Biostatistics, School of Public Health, Wuhan University, Wuhan, Hubei China; Global Health Institute, Wuhan University, Wuhan, Hubei China; Department of Social Medicine and Health Management, School of Public Health, Wuhan University, Wuhan, Hubei China

## Abstract

**Background:**

Breast cancer is the most common cause of cancer—related death among women. In this paper, we studied the variations in the trends of Chinese female breast cancer mortality by age, period and cohort from 1990 to 2009.

**Methods:**

The mortality data were collected from the Institute for Health Metrics and Evaluation. An age-period-cohort model and Intrinsic Estimator were used to estimate the age effect, period effect and cohort effect on the Chinese adult female breast cancer mortality risk.

**Results:**

The age effect on Chinese female breast cancer mortality initially increased, but then declined, and showed a reversed “J” shape with age. The period effect of breast cancer mortality continued to rise with the time period and increased by 59 % from 1990–1994 to 2005–2009. The cohort effect showed that the mortality risk of Chinese females born after 1911 was on the decline and decreased by 2.2336 from 1911 to 1989. The change rate of the cohort effect on breast cancer mortality fluctuated regularly. Three accelerating decreases and three decelerating decreases were noted in the variation law of the change rate.

**Conclusion:**

The results of study show the increasing mortality trend of breast cancer in Chinese female from 1990 to 2009, and the breast cancer mortality risk decreased with birth cohort.

**Electronic supplementary material:**

The online version of this article (doi:10.1186/s12939-015-0211-x) contains supplementary material, which is available to authorized users.

## Introduction

Cancer has become a serious public health challenge worldwide. As the most common cancer in females, breast cancer has an impact on the everyday lives of women and is a common cause of death. According to the Globocan [1] data published by the International Agency for Research on Cancer (IARC), almost 1.67 million new breast cancer cases were diagnosed in 2012, which accounted for 25 % of all cancer cases in women. Additionally, approximately 522,000 deaths from breast cancer were reported in 2012, which represents 14.7 % of all cancer deaths in women. In China, there were 187,000 new breast cancer female cases and 48,000 female deaths in 2012.

Mortality rate is one of the most important indicators for monitoring the health status of breast cancer patients. Breast cancer has been the main cause of death in many regions worldwide, and the mortality rates of breast cancer have increased among Chinese females. According to the results of three death cause surveys, the breast cancer mortality of female showed an upward tendency. The standard mortality for Chinese female increased 36.1 % from 1970s to 2005, while it decreased obviously in Europe and America developed countries.

With the increasing breast cancer burden in China, the study on the trend of breast cancer mortality has become more and more. However, there are many studies failed to analysis the cohort effect or apply the flawed methods about the parameter estimation of Age-period-Cohort Model. For instance, Zheng Y [2] and Ma S[3] only studied the time trends and distribution characteristics of female breast cancer mortality. Wang YH [4] applied age-period-cohort model and coefficient constrained approach to study the influences on breast cancer mortality which was reflected by the effects of age, period and cohort.

In this paper, we examined the trends in Chinese female breast cancer mortality by age, period and cohort. A statistical analysis of the breast cancer mortality of 20–79-year-old Chinese females from 1990 to 2009 was performed. The age effect, period effect and cohort effect were estimated by an Age-Period-Cohort (APC) model combined with the Intrinsic Estimator algorithm. APC modeling allowed us to use the present mortality rates to analyze the variations in the tendencies and the regular patterns of mortality in the past few decades or over the past century, even when data were missing or inaccurate.

Studying the trends in Chinese female breast cancer mortality may reveal new information about the risk factors for breast cancer. The results of the period effect and cohort effect could reveal the relationship between social development and breast cancer burden.

## Methods

### Data source

The national female breast cancer (coded in the ICD-10) mortality rates were extracted from the Institute for Health Metrics and Evaluation (IHME, http://ghdx.healthdata.org/). The IHME is an independent global health research center at the University of Washington in the US. The IHME Global Health Data Exchange combines registry data, surveys, censuses and other health-related data to produce mortality estimates. The IHME includes many scientists from dozens of countries in publishing the Global Burden of Diseases, Injuries and Risk Factor Study. With reference to China, the data were collected from Vital Registration, the Ministry of Health, China Vital Statistics-Deaths, the Cancer Registry, and the WHO Mortality Database, among other sources. The Gaussian process regression (GPR) was used to adjust the raw data and derive trend estimates by the IHME. Therefore, the data are highly reliable.

The statistics for a period of 5 years are required to make an APC model. After excluding Chinese females under 20 years old and above 80 years old, the data used in our study were from the age groups 20–24 years old to 75–79 years old. The time horizon of data started from 1990–2009 (with 5 years per period). The age-specific and period mortality rates were computed.

### Age-period-cohort model

The APC model represents a classic epidemiological method that can be used to extract information from cross-sectional data regarding historical changes in the morbidity and mortality risk, termed the cohort effect [5]. As there is a linear relationship between the age, period and cohort, it is difficult to estimate the unique set for every age, period and cohort effect, which is referred to as the un-identification problem [6]. Although various statistical methods have been used to address this issue, many researchers still do not agree on methodological solutions to this problem [7–10]. The IE algorithm is a new and promising method to solve the un-identification problem associated with the APC model. It has since proven that unique solutions can be computed using the APC model with the IE algorithm [11, 12].

### Intrinsic estimator method

Fu [13] applied the estimable functions and the singular value decomposition of matrices to approach the estimator of the APC model, which he named the Intrinsic Estimator (IE). So why could the IE  solve the identification problem? As noted by Fu [12], there have been only numerical demonstrations to support the idea that no estimable function exists, but no rigorous evidence. Kupper et al [14] provided a condition for estimable functions and suggested that an estimable function satisfying this condition resolves the identification problem. The IE satisfies this condition and estimates the unique estimable function(Additional file 1).

In this paper, all analyses were implemented using the Stata version 12.0 software program (StataCrop LP, Texas, USA). Fitting deviance, the Akaike Information Criterion (AIC) and the Bayesian Information Criterion (BIC) were used to evaluate the model.

## Results

### Variation trends of age, period and cohort on breast cancer mortality

**Fig. 1:**
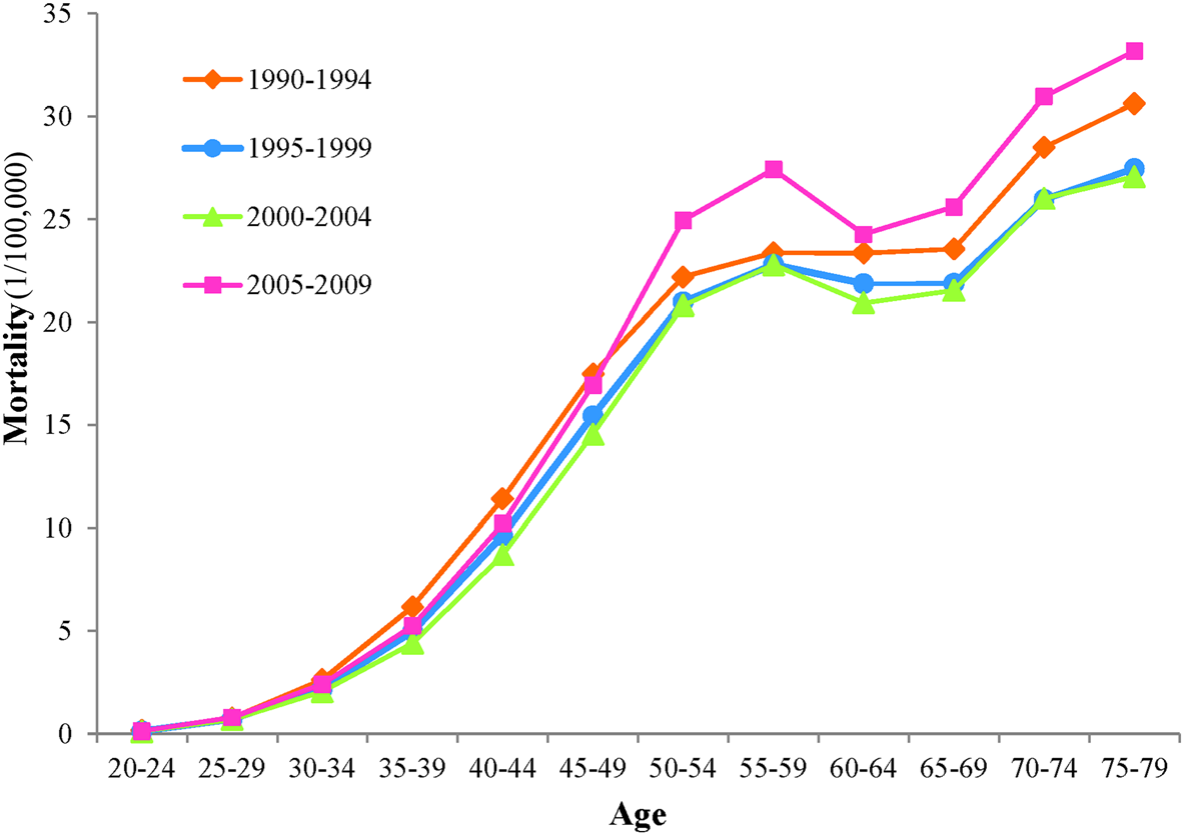
Age-specific breast cancer mortality in different periods among Chinese females (per 100,000 people). The breast cancer mortality rates of different age groups (20–24 years old to 75–79 years old) in each period (1990–1994, 1995–1999, 2000–2004, 2005–2009) are shown

The variation trends in the age-specific breast cancer mortality among Chinese females aged 20–79 during 1990–2009 are shown in Fig. 1. Regardless of the time period, the breast cancer mortality generally increased with age, and the breast cancer mortality rates increased at a steady rate before the subjects reached 55 years old. However, breast cancer mortality decreased in the group aged 60–64 years old, and then continued to increase again from 65 years old. From the change trend in the period perspective, the breast cancer mortality rates of all age groups in the periods 1990–1994, 1995–1999 and 2000–2004 presented a decrease with the time period, except for 2005–2009. In particular, for the patients 50 years old and older, the breast cancer mortality rates of all age groups in the time period 2005–2009 were higher than those of the other three periods.

**Fig. 2:**
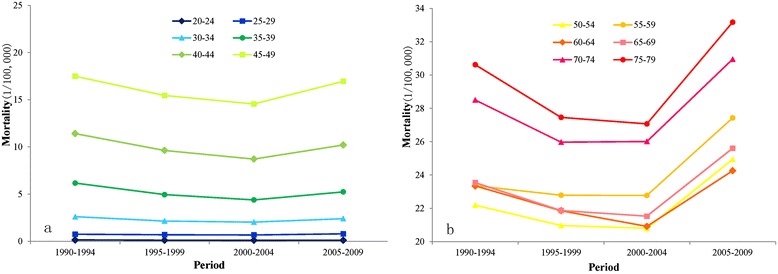
Breast cancer mortality of different age groups among Chinese females (per 100,000 people). The breast cancer mortality rates of the different periods (1990–1994, 1995–1999, 2000–2004, 2005–2009) in each age group (20–24 years old to 75–79 years old) are shown. Figure 2 (a) shows the breast cancer mortality of 20- to 49-year-old females, and Figure 2 (b) shows the breast cancer mortality of 50- to 79-year-old females.

The variations in the breast cancer mortality rates of different age groups during the decades from 1990–2009 are shown in Fig. 2 (a, b). Figure 2 (a) shows the breast cancer mortality of 20- to 49-year-old females, while Fig. 2 (b) shows the breast cancer mortality of 50- to 79-year-old females. The breast cancer mortality of all age groups decreased first and then increased with the time period. Except for the 70- to 74-year-old group, which showed an increase during the period from 1995–1999, the breast cancer mortality of the other age groups increased during the period from 2000–2004. In general, as the age group became older, the breast cancer mortality rate increased during the entire study period (1990–2009), and increases were also observed specifically for the 60- to 64-year-old and 65- to 69-year-old groups. The breast cancer mortality rates of these two groups were between those of the 55–59 and 50- to 54-year-old groups from 1990–2004. However, the breast cancer mortality of the 60- to 64-year-old group was even lower than that of the 50- to 54-year-old group in the period from 2005–2009.

### APC model analysis results of breast cancer mortality

The APC model was used to analyze the age-specific breast cancer mortality, and the age effect, period effect and cohort effect were calculated by the IE algorithm. The results of the analysis of breast cancer mortality are shown in Table 1.

**Table 1:** APC model analysis results of Chinese female breast cancer mortality

Age	Coef.	SE	Period	Coef.	SE	Cohort	Coef.	SE
20–24	−3.1995	1.3652	1990–1994	−0.1659	0.1020	1911–1919	0.9986	0.3124
25–29	−1.6295	0.5876	1995–1999	−0.1219	0.0705	1916–1924	0.8498	0.2567
30–34	−0.6523	0.4214	2000–2004	−0.0136	0.0721	1921–1929	0.7206	0.2256
35–39	−0.0240	0.3465	2005–2009	0.3013	0.0958	1926–1934	0.6106	0.2044
40–44	0.4461	0.2927				1931–1939	0.4693	0.2154
45–49	0.7539	0.2446				1936–1944	0.3441	0.2370
50–54	0.9254	0.2001				1941–1949	0.2279	0.2669
55–59	0.8764	0.1636				1946–1954	0.1151	0.3024
60–64	0.6887	0.1396				1951–1959	−0.0437	0.3427
65–69	0.5896	0.1351				1956–1964	−0.2539	0.3875
70–74	0.6490	0.1504				1961–1969	−0.4601	0.4366
75–79	0.5761	0.1859				1966–1974	−0.6310	0.4993
						1971–1979	−0.7809	0.6300
						1976–1984	−0.9314	0.9932
						1981–1989	−1.2350	2.9850
Intercept				2.013				
Model fitting								
Deviance	0.2488		AIC	5.1486		BIC	−77.1752	

According to the analysis of the age effect (Table 1), the age effect on breast cancer mortality essentially showed a reversed “J” shape with age. The group of patients aged 20–24 years old had the lowest breast cancer mortality risk. The mortality risk of breast cancer initially increased rapidly to 0.9254 (peak value) from the 20- to 24-year-old group to the 50- to 54-year old group, and then decreased to 0.5761 in the group aged 75–79 years old. To interpret the age effect more intuitively, the coefficients were transformed into relative risk (RR). The relative risk of 50-to 54- year-old group for breast cancer death were 61.86 (*RR* = *e*^[0.9254 − (−3.20)]^) and 1.61 (*RR* = *e*^[0.9254 − 0.4461]^) compared with the groups of subjects 20–24 years old and 40–44 years old, respectively.

The period effect of breast cancer mortality showed a net increase of 0.4672 = [0.3013-(−0.1659)] from the period of 1990–1994 to the period of 2005–2009 (Table 1). Similarly, the relative risk of period group 2005–2009 were 1.60 (*RR* = *e*^[0.3013 − (−0.1659)]^), 1.53 (*RR* = *e*^[0.3013 − (−0.1219)]^) and 1.37 (*RR* = *e*^[0.3013 − (−0.0136)]^) compared with period group 1990–1994, 1995–1999 and 2000–2004, respectively. The period effect alone increased the breast cancer mortality risk by 59.55 % over a period of 20 years, indicating an annual average growth of 2.98 %.

The cohort effect of breast cancer mortality in Table 1 showed the variations in breast cancer mortality risk of Chinese females born in different birth years. Obviously, the mortality risk of Chinese female born after 1911 was on the decline. The breast cancer mortality risk decreased by 2.2336 = [0.9986-(−1.2350)] from birth cohort 1911–1919 to 1981–1989. Using the birth cohort 1911–1919 as a benchmark, the cohort effect of breast cancer mortality was reduced by 21.05 % after the data were standardized and converted. Because of the decline trend of cohort effect, we can see that the relative risk of birth cohort 1981–1989 was 0.11 (*RR* = *e*^[−1.235 − 0.9986]^) compared with the oldest birth cohort 1911–1919.

### Change rate of cohort effect on breast cancer mortality

According to the mathematical meaning of numerical differentiation, we calculated the change speed of the cohort effect by numerical differentiation. The differential value is positive if the mortality risk is increased, negative if the mortality risk is decreased and 0 if the mortality risk remains the same.

**Fig. 3:**
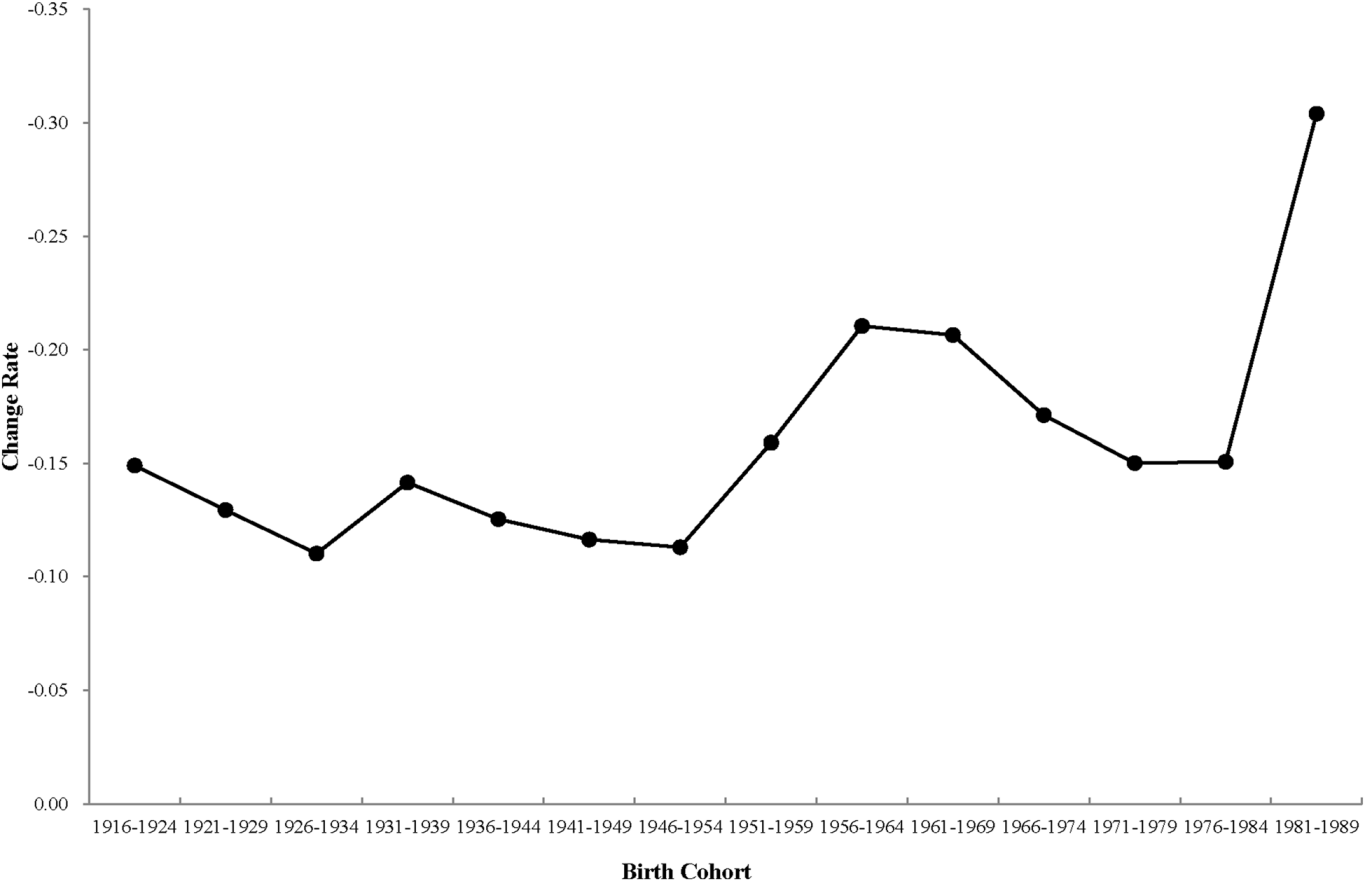
Change rate of the cohort effect on breast cancer mortality during the period from 1911–1989. The velocity of the change in the cohort effect on Chinese female breast cancer mortality from the birth cohort of 1911–1919 to 1981–1989 is shown

As shown in Fig. 3, the change rates of the cohort effect on breast cancer mortality fluctuated from 1916 to 1989. The scale value of the Y-axis was reversed. The figure shows that there were broken lines above the X-axis and all of the change rates were negative, which means that the mortality risk of breast cancer decreased. The larger absolute value for the change rate indicates a faster variation of the mortality risk. Therefore, where the line rises, the change rate of the mortality risk accelerated, or conversely, decelerated.

As shown in Fig. 3, there were three accelerating decreases and three decelerating decreases. The three accelerating decreases were from 1926–1934 to 1931–1939, 1946–1954 to 1956–1964, and 1971–1979 to 1981–1979, respectively. The change rate of mortality accelerated by 0.0313, 0.0974, and 0.1537, respectively. The other three sections were decelerating decreases.

## Discussions

The APC model and the Intrinsic Estimator algorithm were used to analyze the variation trends of breast cancer mortality among Chinese females aged 20–79 years during the period from 1990–2009 in this study. Because of the collinearity of three factors (age, period and cohort) and the un-identification problem, there are many statistical methods used to solve this problem, e.g., Constrained Generalized Linear Models, Two Factor Models, Nonlinear Models, Penalty Function Approach and Estimation Function, et.al. Compared with these methods, the IE algorithm could obtain the unique solution of parameters without any constrained conditions or new variable. In addition, the estimated values are interpreted more intuitively and unbiased. To verify the validation of IE estimates, Monte Carlo simulations are conducted for the IE and other conventional coefficients constrained approaches. It is proved that the MSEs of the IE estimates are much smaller and close to zero.

The age effect of breast cancer mortality among Chinese females aged 20–79 years old essentially showed a reversed “J” shape with age. This trend indicated that the older the age, the higher the breast cancer mortality. Compared to the subjects 20–24 years old, the mortality risk of subjects 50–54 years old increased by 61.86 %, while the mortality risk of the patients 75–79 years old increased by 43.62 %.

According to the analysis of the period effect on breast cancer mortality, there was a net increase of 0.4672 from the period from 1990–1994 to that from 2005–2009, indicating an annual average growth of 2.98 %. Such rapid growth may suggest that environmental deterioration and unhealthy lifestyle behaviors such as increased alcohol consumption, obesity, and a lack of physical exercise, increase the period effect of breast cancer mortality risk during these time periods [15, 16]. In other words, the deterioration of the environment and an unhealthy lifestyle caused by economic development might have increased the period effect on the breast cancer mortality risk of Chinese females aged 20–79 years old and aggravated the breast cancer—related mortality over time.

Although the cohort effect on the breast cancer mortality of Chinese females born after 1911 was on the decline, the change rate of the mortality risk fluctuated regularly. Three accelerating decreases and three decelerating decreases were noted for the change rate of the mortality risk. The accelerating decreases in the change rate represent improvements in the breast cancer mortality, while the decelerating decreases in the change rate represent a relative deterioration of breast cancer mortality. The three accelerating decrease started from 1926–1934 to 1931–1939, 1946–1954 to 1956–1964 and 1976–1984 to 1981–1989, respectively. The three decelerating decreases were from 1916–1924 to 1926–1934, 1936–1944 to 1941–1949 and 1961–1969 to 1971–1979, respectively.

Changes in lifestyle and dietary habits may have led to the increasing trends in breast cancer mortality. Kaizer [17] analyzed the relationship between dietary fat and breast cancer for 32 countries and discovered that a high intake of dietary fat has a significant positive correlation with breast cancer mortality. Hamajima [18] reported that the relative risk of breast cancer increased by 7.1 % (95 % CI 5.5–8.7 %; P < 0.00001) for each additional 10 g per day intake of alcohol, and the degree of growth was the same for the never-smokers and ever-smokers. Smoking, as well as passive smoking, has probabilities to influence female of all ages, and has been known as a risk factor for female breast cancer [19]. Three nationwide epidemiology surveys about smoking were carried out in the year of 1984, 1996 and 2002, respectively. The prevalence of smoking in China remains at a high level. Although smoking rates decreased from 1984 to 2002, the numbers of smoking increased 100 million, and the people who smoke tends to be younger [20]. Especially for female, smoking rates of the groups aged 15–19 and 20–24 showed the obviously ascending trend [21]. In addition, an estimated 72 % of Chinese individuals over the age of 15 years had been exposed to tobacco, including those exposed to second-hand smoke [22].

On the other hand, many pathological and epidemiological studies have found that benign breast lesions (BBL) also have a relationship with breast cancer [23]. Liu reported that BBL are associated with the risk of breast cancer, and the OR was 4.1 (95 % CI 2.5–6.4) in China [16]. Thus, breast cancer screening is very important for Chinese female breast health. In addition, studies of medical psychology have proven that anxiety, stress and depression can increase the risk of breast cancer [24].

Additionally, many studies have indicated that an early age at menarche, late menopause age and fertility are associated with breast cancer risk [25–28], and long-term breast feeding has been suggested to reduce the risk of breast cancer in China [29, 30]. Unfortunately, there are no statistical data concerning the change in the age at menopause age and breastfeeding over such a long period in China. However, Zhao studied the changes in trends of early menarche in China from 1985 to 2005 and discovered that the trend of early menarche slowed or even stagnated [31]. Another study showed that the total fertility rate increased 9.5 % from 2000 to 2005, and then fell from 1.338 in 2005 to 1.188 in 2010 [32].

Thus, breast cancer screening, detection, and changes in dietary habits are associated with breast cancer risk [30, 33]. In particular, breast cancer screening and dietary factors are related to the period effect of breast cancer mortality. As there is currently no breast cancer screening program in China, the National Health and Family Planning Commission (NHFPC) performed breast cancer screening nation-wide in 2008 and found that the awareness and participation rate of screening for breast cancer in Chinese females are very low [33]. In addition, the detection rate of breast cancer is lower than in Western countries [34]. This imperfect breast cancer screening and the lower detection rate make it difficult to obtain an early diagnosis and treatment, which lead to increased breast cancer mortality rates. The proportion of fat from animal-based foods in the diet also increased 70.73 % from 1985 to 2001 [35]. The status and changes of breast cancer screening, the breast cancer detection rate, and changes in dietary habits likely acted in combination to increase the breast cancer morality in China, thus supporting the period effect on breast cancer.

A major limitation of the present study was lack of analysis on incidence of breast cancer Chinese female. The incidence is one of the important factors which influenced the trend of breast cancer death. According to the Cancer Registry, the crude incidence of breast cancer for Chinese female increased 1.2–2.8 times, and the standardization incidence increased 4.0–7.3 times during 1988–2007 [36]. Thus, a deeper analysis combined with incidence will be studied in future. Furthermore, as lacking knowledge about the comprehensive effects of the risk factors of breast cancer in China, some results from age-period-cohort models should be interpreted with caution.

In summary, although the cohort effect of breast cancer decreased, the mortality rate of breast cancer increased during the period from 1990–2009. The morality pattern of breast cancer in China can be explained by the period effect. The imperfect breast cancer screening, low detection rate and unhealthy dietary habits have likely all played an important role on the period effect of breast cancer mortality among Chinese females.

## Additional file

Additional file 1:
**Supplemental method.** (DOCX 69 kb)
