# Supplementary material for: An age-period-cohort analysis of female breast cancer mortality from 1990–2009 in China
Source: Int J Equity Health. 2015 Sep 14;14:76. doi: 10.1186/s12939-015-0211-x (PMC4568582; doi:10.1186/s12939-015-0211-x)
Supplement: Additional file 1: — Supplemental method. (DOCX 69 kb) [file 12939_2015_211_MOESM1_ESM.docx]

Supplemental method

Intrinsic Estimator Method

APC model can be written as the form of linear regression, for the mortality, the model should be:

$$M_{ij}=\mu+\alpha_{i}+\beta_{j}+\gamma_{k}+\varepsilon_{ij}\text{ }\left(1\right)$$

where $M_{ij}$ is the mortality for the *i*th age group at the *j*th period of observed data, $i=1,2,\cdots\cdots,a$,$j=1,2,\cdots\cdots,p$; $\mu$ is the intercept or adjusted mean mortality; $\alpha_{i}$ is the age effect or the coefficient of the *i*th age group; $\beta_{j}$ is the period effect or the coefficient of the *j*th period ; $\gamma_{k}$ is the cohort effect or the coefficient of the *k*th ($k=a-i+j$) cohort, $k=1,2,\cdots\cdots,a+p-1$; and $\varepsilon_{ij}$ is the a random error with expectation $E(\varepsilon_{ij})=0$.

Since there is a linear relationship between age, period and cohort, that is,

$$period=age+cohort\text{ }(2)$$

It leads a un-identification problem for model (1).

For model (1), it can be treated as fixed-effects generalized linear models after a reparameterization to center the parameters:

$$\sum_{i} \alpha_{i}=\sum_{j} \beta_{j}=\sum_{k} \gamma_{k}=0\text{ }(3)$$

After this reparameterization, model (1) can be written in the matrix form:

$$Y=Xb+\varepsilon\text{ }(4)$$

where $Y$is a vector of mortality rates or log-transformed rates, $X$ is the regression design matrix consisting of “dummy variable” column vectors for the vector of model parameters $b$:

$$b=(\mu,\alpha_{1},\cdots\cdots,\alpha_{a-1},\beta_{1},\cdots\cdots,\beta_{p-1},\gamma_{1},\cdots\cdots,\gamma_{a+p-2})^{T}\text{ }(5)$$

Here, $T$ means the transposition of the vector, that is, transform column vector to row vector.

In order to estimate the unique parameter$b$, the theory of algebra will be used. The parameter space of the unconstrained APC regression model (4) can be decomposed into the direct sum of two linear subspaces that are perpendicular to each other. One subspace corresponds to the unique zero eigenvalue of the matrix $X^{T}X$ and is of dimension 1; it is termed the null subspace of the design matrix$X$. The other, non-null subspace is the complement subspace orthogonal to the null space.

So the form of could be written as:

$$\hat{b}=B+sB_{0}\text{ }(6)$$

where $s$ is a scalar corresponding to a specific solution and $B_{0}$ is a unique eigenvector of Euclidean norm 1. The eigenvector $B_{0}$ does not depend on the observed rates *Y*, only on the design matrix *X*, and thus is completely determined by the numbers of age groups and period groups—regardless of the event rates. And $B_{0}$belongs to null subspace of the design matrix$X$, and satisfies$XB_{0}=0$.

In equation (6), $B$is the special estimator termed the IE that lies in the parameter subspace that is orthogonal to the null subspace.

As to the estimation of an initial estimator$\hat{b}$, Stata Software could realize the computation. The users of Stata can install a software package by typing “*ssc install apc*” on the command window on the computer connected to the Internet. If the dataset contains a dependent variable $y$(e.g., mortality or other rates), an age variable $a$and a period variable$p$, the users can fit a Poisson model with age, period and cohort effects by typing

$$apc\_ie\text{ }y,age(a)\text{ }period(p)\text{ }family(poisson)\text{ }link(\log)$$

where log-linear regression is used.
